# Effect of tamarind (*Tamarindus indica* L.) seed on antioxidant activity, phytocompounds, physicochemical characteristics, and sensory acceptability of enriched cookies and mango juice

**DOI:** 10.1002/fsn3.311

**Published:** 2015-11-18

**Authors:** Sheilla Natukunda, John H. Muyonga, Ivan M. Mukisa

**Affiliations:** ^1^ School of Food Technology, Nutrition & BioengineeringMakerere UniversityP.O. Box 7062KampalaUganda

**Keywords:** Antioxidant activity, condensed tannin, flavonoid, phenolics, phytocompounds, tamarind seed

## Abstract

Tamarind seeds are not consumed despite their high antioxidative activity. In this study, 0–10% tamarind seed powder (TSP) was incorporated into mango juice and cookies. Total phenolics (Folin–Ciocalteu assay), antioxidant activity (2,2‐diphenyl‐1 picrylhydrazyl (DPPH) radical scavenging assay), flavonoid (aluminum chloride assay), condensed tannins content (Vanillin‐HCl assay), and consumer acceptability (*n* = 50) of the products were determined. TSP increased the pH and viscosity and reduced titratable acidity of juice. Incorporation of TSP increased the: total phenolic content (6.84 ± 0.21 to 88.44 ± 0.8 mg GAE/100 mL); flavonoid (4.64 ± 0.03–21.7 ± 0.36 mg CE/100 mL); condensed tannins (0.24 ± 0.01–21.81 ± 0.08 mg CE/100 mL) and total antioxidant activity (4.65 ± 0.88–21.70 ± 0.03 mg VCE/100 mL) of juice. A similar trend was observed for cookies. Maximum sensorially acceptable TSP levels were 1.5% and 6%, respectively, for juice and cookies. TSP can thus be utilized as a source of natural antioxidants in food products.

## Introduction

Epidemiological studies have consistently demonstrated that consumption of plant‐derived foods rich in bioactive phytochemicals has a protective effect against oxidation stress (Librandi et al. 2007; Ovaskainen et al. 2008; Galili and Hovav 2014). Oxidative stress is strongly associated with mutagenesis, carcinogenesis (Abnet et al. 2015), aging (Everitt et al. 2006), and atherosclerosis in humans. The bioactive phytochemical compounds thus decrease the risk of chronic diseases, cardiovascular disease, cancer, and degenerative diseases of the aging (Keservani et al. 2010).

Currently, industries are interested in developing value‐added products from the waste‐by products generated by both the food and agricultural processing industries (Balasundram et al. 2006). The waste products including seeds, peels, stalks, stems, and leaves of plants contain substantial amount of phenolics and thus can be used as cheap sources of natural antioxidants for pharmaceutical, cosmetic, and food application (Bucic´‐Kojic´ et al. 2009). Fruits and vegetable waste products including seeds have been reported to have higher content of bioactive phytochemicals than the edible portions (Soong and Barlow 2004). Fruit seeds contain a variety of biologically active phytochemical compounds, especially phenolic constituents, flavonoids, anthocyaninins, vitamin C, and carotenoids. These phytochemicals positively influence human health and indicate high antioxidant activity (Pérez‐Jiménez et al. 2008). Hence, it is considered crucial to increase the antioxidant intake in the human diet and one way of achieving this is can be through enriching food products with seeds which are rich in phytochemicals.

Tamarind (*Tamarindus indica*) seed is a by‐product in the tamarind pulp industry. Recently, a large amount of the seed waste is discarded from the tamarind industry (Oluseyi and Temitayo 2015). Tamarind seed is a rich source of phytochemicals (Tsuda et al. 1994; Andabati and Muyonga 2014) which consists of phenolic antioxidants, such as 2‐hydroxy‐3′,4′‐dihydoxyacetophenone, methyl 3, 4‐dihydroxybenzoate, 3,4‐dihydroxyphenyl acetate and epicatechin (Sudjaroen et al. 2005; El‐Siddig et al. 2006). Tamarind seed extracts exhibit antioxidant potential by reducing lipid peroxidation in vitro (Tsuda et al. 1994) and anti‐microbial activity. Tamarind seed therefore has the potential of providing low cost nutritional and nutraceutical value.

In this study, tamarind seed powder (TSP was evaluated as a source of antioxidants for inclusion in two commonly consumed products; cookies and mango juice. Cookies and mango juice are regularly consumed by nearly all age groups in developing countries. Cookies are popular compared to other processed foods because of their low cost, diverse taste, myriad shapes, and long shelf life (Davidov‐Pardo et al. 2012). Jesionkowska et al. (2009) reported that cookies were selected by consumers as a good vehicle for antioxidants. Cookies also contain fat which possess' sensory characteristics that are ideal to mask the astringent flavor and taste associated with tamarind seed which is undesirable for most consumers.

Mango (*Mangifera indica)* is the most vital and widely cultivated among the tropical and subtropical fruits (Akhter et al. 2012). Thus, mango juice was selected because of the abundance, seasonal availability, popularity, and distinctive flavor and taste. Mangoes also have a strong flavor and substantial levels of pectin which can mask the astringency of the tamarind seed without affecting antioxidant activity (Laaksonen 2011; Soultani et al. 2014).

While tamarind seeds are known to contain substantial levels of bioactive phytocompounds, it was not clear if they could be used to enhance nutraceutical properties of widely consumed processed foods while retaining product acceptability. This study was therefore undertaken to assess the effect of tamarind seed powder on the physicochemical properties, phytonutrient content, antioxidant, and sensory properties of the enriched selected food products.

## Material and Methods

### Materials

Fresh tamarind fruits were purchased from the local markets in Mbale district, Uganda and transported to the Department of Food Technology and Nutrition, Makerere University. Fresh and fully ripened mangoes were obtained from the local market in Kampala, Uganda and transported to the Department of Food Technology and Nutrition, Makerere University. The fruits were selected for uniformity in shape and color, washed carefully with clean portable water and stored in a refrigerator at 8^°^C prior to further use.

Commercial wheat flour (Bakhresa Grain Milling (U) Ltd, Uganda), sugar (Kakira Sugar Works, Jinja, Uganda) and sodium bicarbonate (Bidco oil Refineries, Nairobi, Kenya) were purchased from the local supermarkets in Kampala Uganda.

### Methods

#### Preparation of tamarind seed powder

Tamarind fruit pods were manually cracked and from each pod the pulp containing seeds was separated along with the fibers. Tamarind seed with fibers were soaked overnight in clean portable water (1: 3 w/v) to enable removal of the pulp and fiber strands. The seeds were then washed using clean distilled water. The cleaned seeds were sun dried in a shaded for 14 days, milled into fine flour using a Wonder mill (Grain of Truth Bread Company, Arlington, VA). The flour was sieved using a 300 *μ*m screen and stored in an airtight container in a freezer at −18°C prior to further use.

#### Mango juice enrichment with tamarind seed powder

##### Preparation of mango juice

The mangoes were rinsed with portable water, peeled, and sliced. Mango juice was then extracted using a blender (Royal Philips, model HR 2167/40, Amsterdam, The Netherlands) and filtered using a sterile cheese cloth. The filtered juice was then blended with clean portable water (1: 3 of mango juice: water) and sugar 3% (w/v) was added to make the desired concentration of 9°Brix. The Mango juice was mixed with TSP powder in different proportions 0%, 0.5%, 1.5%, 1%, 2% and 2.5% (w/v) using a portable hand mixer (sun beam model SHM 100, Nu world industries Ltd, 2000).

##### Pasteurization treatment of tamarind seed mango juice and storage conditions

The tamarind seed and mango juice blend of 600 mL in a 1 L capacity container was pasteurized at 85°C with a holding time of 25 sec (Moyer and Aitken 1980). The beaker containing the juice was placed in a water bath maintained at 95°C and the juice was heated for 20 min while stirring until it reached 85°C. It was maintained at this temperature for 25 sec before hot filling in amber plastic bottles. The enriched juice was pasteurized to enable extraction of phytochemical compounds as well as to destroy spoilage microorganisms(Andabati and Muyonga 2014). The juice was previously filtered with a clean sterile cheese cloth before hot filling in amber plastic bottles. The amber bottles containing juice were capped and cooled to room temperature in a water bath. The bottles with pasteurized juice were stored in the refrigerator at 8°C subsequent to further analyses.

#### Cookie enrichment with tamarind seed powder

##### Preparation of flour mixtures and baking of enriched cookies

Cookies were prepared according to the method number 10–50D AACC (2000) with some modifications in the recipe (Table 1). Control samples were prepared without addition of TSP. TSP‐enriched cookies were prepared by substituting wheat flour with of 2%, 4%, 6%, 8% and 10% TSP. Tamarind seed powder was well blended with wheat flour, sugar, and fat. The sugar and fat were creamed manually. The baking flour, baking powder, and TSP were sieved together and added to the cream before mixing it uniformly. The cookie dough was rolled, sheeted to a thickness of 3.5 mm and cut using a circular mould (5 cm diameter). Baking was done at 150°C for 40 min. After baking, cookies were cooled to room temperature and packed into airtight polythene bags until further analysis.

**Table 1:** Formulation for tamarind seed‐enriched cookies

Formulation description	Wheat flour (g)	Sugar (g)	Fat (g)	TSP (g)	Egg	Baking powder (g)
Control	250	80	100	0	1	3
2% TSP	245	80	100	5	1	3
4% TSP	240	80	100	10	1	3
6% TSP	235	80	100	15	1	3
8% TSP	230	80	100	20	1	3
10% TSP	225	80	100	25	1	3

#### Physicochemical analyses

##### pH

The pH of TSP‐enriched mango juice samples and control was measured with a pH‐meter (pH meter, Hanna instrument H12210, Crison Instruments S.A., Barcelona, Spain) at room temperature (~25°C) according to AOAC official method 943.02 (1995). Buffer solutions of pH 4.0 and 7.0 were used for periodical calibration of pH meter. Three readings were performed for each replicate.

##### Color

The color of the juice was determined using a color tintometer (Lovibond Model E, AF‐900; Tintometer Limited, Salisbury. U.K.). The juice was filled in the one inch optical glass cell which was placed into the cell channel. Sample colors were matched by a suitable combination of the three primary colors together with neutral filters using a viewing tube and adjusting the color filter knobs. The resulting set of Lovibond red, blue, yellow, and neutral (RYBN) units were used to define the color of the mango juice and TSP‐enriched mango.

##### Viscosity measurements and analysis

Viscosity of the tamarind seed‐enriched mango juice and control juice was measured using a Brookfield Viscometer (Brookfield LVDV‐II+P, Brookfield Engineering Laboratories, Inc., Middleboro, MA). TSP‐enriched mango juice of 500 mL was loaded into a glass beaker reservoir (cylindrical shape) of 600 mL capacity and was allowed to equilibrate at room temperature. Spindle LV‐ 1 was used to measure viscosity of juice, using rotational speeds ranging between 0.3 and 1.5 revolutions per minute (RPM). The recording of the viscometer output commenced 3 min after the onset of the experiment.

##### Total soluble solids

Total soluble solids content of the tamarind seed‐enriched mango juice and control was determined with a handheld refractometer (Westover Model RHB‐32; Southwest United Industries, Tulsa, OK) using AOAC (1999) method 981.12. The results were reported as ^o^Brix at 20°C.

##### Titratable acidity

The total titratable acidity of the juices was determined according to AOAC (1999) by titration, using 0.1N sodium hydroxide with phenolphthalein. The results were expressed as % tartaric acid, using the weight of the molar mass of tartaric acid as the equivalent weight of acid (Banigo and Muller 1972).

##### Quantification of total carotenoids

Total carotenoids in tamarind seed powder‐enriched mango and control juice was determined according to the method of Rodriguez‐Amaya and Kimura (2004). Briefly, 2 mL of TSP‐enriched mango juice was extracted by mixing with 50 mL cold acetone in the dark. The acetone was removed through the slow addition of 250 mL double distilled water to prevent the formation of emulsions. The aqueous phase was discarded and this procedure was repeated four times until there was no residual no residual acetone. The extract was then transferred through a funnel into a 50 mL volumetric flask containing glass wool with 15 g of anhydrous sodium sulfate. The final volume was adjusted with petroleum ether. Absorbance was measured at 450 nm (Genesys 10‐UV spectrophotometer, Thermo Electron Corporation, Madison, WI) against petroleum ether as a blank. The total carotenoid content was calculated using the following formula:

$$\text{Total carotenoid content}\;\left(\mu \text{g/mL}\right)\;=\;\frac{\text{Absorbance}\times \;\text{Total volume of extract}\times \;10^{4}}{\text{Absorption coefficient of beta carotene}(2592)\times \;\text{sample volume}(mL)}$$

##### Phytochemical analysis

Tamarind seed‐enriched mango juices and cookies including controls (without TSP) were analyzed for total phenolic content, total condensed tannins, total flavonoid content and total antioxidant activity.

##### Extraction of phenolics

The extraction method described by (Makkar 2000) was used with slight modifications. Briefly, 100 mg of TSP‐enriched cookies and control was extracted, using 5 mL of 50% methanol: 50% water solution (v/v). The falcon tube containing the mixture was suspended in ultrasonic water (Bransonic series, M 2800‐E; Branson Ultrasonics Co, Danbury, CT) and subjected to ultrasonic treatment for 20 min at room temperature. The extract was immediately cooled at 4°C in a freezer for 10 min and then centrifuged at 3000 g for 10 min using a centrifuge (Fischer scientific 225, Fisher Scientific Co. St. Louis, MO). The supernatant was collected into a separate tube and stored at 4°C. The pellet was then further re‐extracted under the conditions previously described to ensure efficient extraction. The two supernatants were pooled in an air tight container and stored in a freezer at 4°C to be used in the determination of total phenolics content (TPC), total antioxidant activity (TAA), total flavonoid contents (TFC) and total condensed tannins (TCT).

#### Determination of total phenolic content of TSP‐enriched products

The total phenolic content of the juices and cookies were determined, using the Folin‐Ciocalteu colorimetric method (Makkar 2000) with some modifications. In brief, 100 *μ*L of the diluted juice (1: 10 of juice to water v/v) or cookie extract was pipetted into a test tube covered with aluminum foil and topped up to 0.5 mL with double distilled water. Subsequently 0.25 mL of Folin–Ciocalteu reagent (1 N) was added followed by 1.25 mL of sodium carbonate (20% w/v) and the mixture homogenized using a vortex. The mixture was then incubated at room temperature for 40 min to allow for color development. Absorbance was measured at 725 nm (Genesys 10‐UV spectrophotometer, Thermo Electron Corporation) against methanol as the blank. The total phenolic content was determined using the standard gallic acid calibration curve with varying concentrations (0.02– 0.125) mg/mL). The total phenolic content was expressed as milligram gallic acid equivalent (GAE)/100 mL of the enriched mango juice and mg GAE/100 mg of the enriched cookies.

#### Determination of total condensed tannins of TSP‐enriched products

Total condensed tannins were determined using the method described by (Sun et al. 1998) with slight modifications. Briefly, 1.5 mL of vanillin solution (4%) w/v was added to 50 *μ*L of diluted juice (1:10 of juice to water v/v) or cookie extract in a test tube. Immediately, 0.75 mL of concentrated HCl was added and the mixture vortexed. The mixture was incubated at room temperature for 10 min to allow for color development. Absorbance was read at 500 nm (Genesys 10‐UV spectrophotometer, Thermo Electron Corporation) with water as a blank. A standard curve was developed using catechin standards of varying concentrations (0.02 to 0.06 mg/mL). Total condensed tannins values were expressed as mg catechin equivalent/100 mL of the juice and mg catechin equivalent/100 g of cookies.

#### Determination of total flavonoids content of TSP‐enriched products

Total flavonoid content was determined using the method of Muanda et al. (2011). In brief, 0.5 mL of catechin standard solution or juice sample or cookie extract was mixed with 2 mL of deionized water and 0.15 mL of sodium nitrite (5% w/v). After 5 min, 0.15 mL of 10% aluminum chloride was added followed by the addition of 1 mL of molar sodium hydroxide after another 6 min. Finally distilled water was used to adjust the total volume to 5 mL and absorbance was read at 510 nm (Genesys 10‐UV spectrophotometer, Thermo Electron Corporation). A standard calibration curve was plotted using different concentrations of catechin (0.002 to 0.125 mg/mL). Total flavonoid content values were expressed in milligram catechin equivalents per 100 mL of juice (mg CE/100 mL) and cookies in mg CE/100 g of enriched cookies.

#### Total antioxidant activity of TSP‐enriched products

The total antioxidant capacity (TAA) of the tamarind seed and products was determined, using the free‐radical scavenging capacity by use of 1, 1‐diphenyl‐2‐picrylhydrazyl (DPPH) (Brand‐Williams et al. 1995) with minimal modification (Stratil et al. 2006). Briefly, 50 *μ*L of the tamarind seed‐enriched mango juice or cookie extract was added to 2.9 mL of freshly prepared 80% ethanol solution of 100 *μ*M DPPH. The mixture was vortexed and allowed to stand for 30 min in the dark at room temperature. Absorbance of the resulting mixture was measured at 515 nm, using Genesys 10‐UV spectrophotometer (Thermo Electron Corporation) against a blank (80% ethanol). The free‐radical scavenging activity of the juice and cookies was calculated as follows

(1) $$\begin{array}{c} \;\text{Scavenging activity}\left(\%\right)\;\;=[1-(\frac{\text{absorbance of sample}}{\text{absorbance of control}})]\times 100 \end{array}$$

The antioxidant content was determined using a standard curve of ascorbic acid (0.1–8 *μ*g/mL). The results were expressed as milligram vitamin C equivalents per 100 mL of tamarind seed mango juice (mg VCE/100 mL) and cookies as mg VCE/100 g of the cookie.

#### Sensory evaluation of tamarind seed‐enriched products

Sensory analysis of tamarind seed‐enriched mango juice involved the participation of 50 untrained panelists who comprised of students from the Department of Food Technology and Nutrition. The recommended minimum number of panelists for assessing sensory acceptability of a product is 50 since a big number best represents the population (Hough et al. 2006). Each individual evaluated eight sensory characteristics (appearance, taste, color, flavor, consistency, mouth feel, sweetness and thickness) of the enriched juices and control. Each sensory attribute was rated on a 9‐point Hedonic scale. The ratings on the 9‐point hedonic scale used were (9 = “like extremely”;8 = “like very much”; 7 = “like moderately”; 6 = “like slightly”; 5 = “neither like nor dislike”; 4 = “dislike slightly”; 3 = “dislike moderately”; 2 = “dislike very much”; 1 = “dislike extremely”) (Carr et al. 1999). Each subject received six samples (unidentified, with randomly assigned three‐digit codes) of each juice (mango juice with tamarind seed and a control). A control juice sample (without tamarind seed) and five samples with different tamarind seed powder formulations (0.5, 1.0, 1.5, 2.0 and 2.5%). The panelists were presented with 50 mL of each juice sample at room temperature under normal lighting conditions. The tamarind seed‐enriched juice and control samples were prepared the day before and stored in a refrigerator. Bottled water was provided to rinse the mouth between tasting samples.

Sensory analysis of tamarind seed‐enriched cookies was done as described above for the juices. However, cookies were evaluated for color, appearance, texture, taste, flavor, and overall quality. The samples comprised a control cookie sample (without tamarind seed) and five samples with different tamarind seed powder formulations (2%, 4%, 6%, 8% and 10%). Cookies were formulated according to the experimental design and prepared the day before the evaluation day and stored at room temperature.

### Data analysis

All experiments were conducted in triplicate. Statistical analysis of the data was performed by analysis of variance (ANOVA), using Student Edition of Statistix 9.0 software (Analytical Software, Tallahassee, FL). A probability value of difference *P* ≤ 0.05 was considered to denote statistical significance. All data are presented as mean values ± standard deviation (SD). Regression analysis was performed to indicate the relationship between total phenolic and/or flavonoid contents and antioxidant activity.

## Results

### Effect of tamarind seed powder on physicochemical properties of mango juice

The addition of tamarind seed powder (TSP) to mango juice significantly (*P* < 0.05) affected the total soluble solids, pH, titratable acidity, and beta carotene of mango juice (Table 2).

**Table 2:** Effect of tamarind seed powder (TSP) on total soluble solids, pH, titratable acidity and beta carotene of mango juice

TSP concentration (%)	TSS (^0^Brix)	pH	%TTA	*β*‐carotene (µg/100 mL)
Control	9.03 ± 0.04^a^	4.7 ± 0.01^a^	0.17 ± 0.01^a^	892.49 ± 11.79^a^
0.5%	9.00 ± 0.00^a^	4.9 ± 0.01^b^	0.16 ± 0.00^a^	563.27 ± 26.73^b^
1.0%	9.00 ± 00^a^	5.36 ± 0.01^c^	0.15 ± 0.00^b^	468.11 ± 8.90^c^
1.5%	9.00 ± 00^a^	5.51 ± 0.20^d^	0.14 ± 0.01^b^	416.67 ± 20.45^d^
2.0%	10.00 ± 00^b^	5.56 ± 0.01^e^	0.12 ± 0.00^c^	272.63 ± 17.81^e^
2.5%	10 ± 00^b^	5.7 ± 0.00^f^	0.09 ± 0.00^d^	195.47 ± 4.45^f^

TTA, titratable acidity. Data are means of triplicate determination ± standard deviation. Mean values in the same column with different superscript letters are significantly different (*P* < 0.05).

The pH ranged from 4.7 to 5.7 and increased with increase in tamarind seed concentration in the order of control <0.5% <1% <1.5% <2.0% <2.5. Total acidity of TSP‐enriched mango juices decreased with increase in TSP. The total carotenoids decreased in the order of control >0.5% >1% >2% >2.5%. Total soluble solids only significantly increased at 2.0% tamarind seed powder concentration.

### Color

The color of mango juice varied at different concentration of tamarind seed powder. The color of mango juices was generally pale yellow to dull orange color on adding TSP. The highest orange color (2.4 and 2.6 units) of the mango juice was observed at 2% and 2.5% concentration, respectively. The juice appeared as yellow/orange with a shade of yellow (2.3 and 1.9) at 2% and 2.5%, respectively. The color of the juice at 0%, 0.5%, 1%, and 1.5% concentration was 1.5, 1.8, 1.8, and 0.9 orange values, respectively, while the shade of yellow was 5.8, 6.0, 5.0, and 2.3 respectively. Tamarind seed‐enriched mango juice of 0.5% and 1% appeared paler than the rest due to a higher yellow shade.

### Effect of TSP on viscosity of mango juice

**Figure 1:**
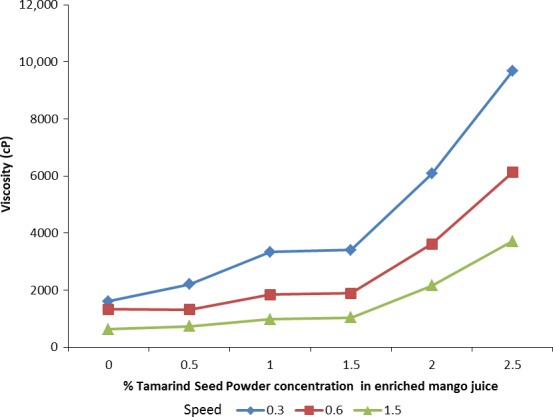
Effect of TSP on viscosity of mango juice.

The viscosity of enriched mango juice increased with increase in tamarind seed powder concentration for all viscometer speeds (Fig. 1). It is clear from Figure 1 that addition of TSP to juices increases the viscosity of enriched juices compared to the control.

### Effect of tamarind seed powder on phytochemical composition of mango juice

Incorporation of tamarind seed powder in mango juice formulation significantly increased the total phenolic, total flavonoids, and total condensed tannin content as well as the total antioxidant activity as compared to the control (Table 3).

**Table 3:** Effect of tamarind seed powder (TSP) on phytochemical composition of mango juice

TSP concentration	TPC (mg GAE 100 mL^−1^)	TFC (Mg CE 100 mL^−1^)	TAA (Mg VCE 100 mL^−1^)	TCT (Mg CE 100 mL^−1^)
Control	6.54 ± 0.21^a^	1.04 ± 0.03^a^	4.64 ± 0.58^a^	0.24 ± 0.01^a^
0.5% TSP	19.50 ± 0.29^b^	8.36 ± 0.06^b^	8.84 ± 0.15^b^	3.59 ± 0.24^b^
1.0% TSP	29.60 ± 0.36^c^	11.87 ± 0.14^c^	13.96 ± 0.28^c^	8.62 ± 0.84^c^
1.5% TSP	43.90 ± 0.14^d^	13.06 ± 0.15^d^	17.91 ± 0.95^d^	11.33 ± 0.29^d^
2.0% TSP	56.06 ± 0.67^e^	17.75 ± 0.28^e^	20.33 ± 0.08^e^	15.99 ± 0.40^e^
2.5% TSP	88.44 ± 0.8^f^	22.48 ± 0.36^f^	21.70 ± 0.04^f^	21.81 ± 0.08^f^

TPC, total phenolic content; TFC, total flavonoid content; TAA, total antioxidant activity; TCT, total condensed tannins. Data are means ± standard deviation from three independent experiments (*n* = 3). Mean values in the same column with different superscript letters are significantly different (*P* < 0.05).

Addition of tamarind seed powder resulted in an increase in the content of total phenolic from 6.54 ± 0.8 to 88.44 ± 0.21^ ^mg GAE/100 mL of the juice. There was a more than 13 fold increase in total phenolic content of enriched juices that received higher TSP (2.5%) as compared to the control.

Mango juice enriched with TSP also showed a similar pattern, with respect to total flavonoid content (TFC), with the value for the 2.5% TSP – enriched juice increased by 20‐fold compared to the control. The total antioxidant activity of the enriched juice increased from 4.64 ± 0.58 for control to 21.70 ± 0.04 mg VCE/100 mL for mango juice containing 3% TSP, while total condensed tannins increased from 3.59 to 21.81 ± 0.08 mg CE/100 mL of the juice.

### Effect of tamarind seed powder on phytochemical composition of cookies

Cookies with tamarind seed powder had significantly higher antioxidant activity and content of the different bioactive compounds (Table 4). Total phenolics ranged from 20.43 ± 0.29 for control to 29.08 ± 0.23 mg GAE/100 g for cookies containing 10% TSP. Tannins content ranged from 8.3 ± 0.73 to 19.24 ± 0.40 mg CE/100 g (Table 4). Enriched cookies that received higher TSP concentration (10%) showed an increase of fivefold and 2.5 fold in both total antioxidant activity and flavonoid content, respectively, as compared to the control juice (without TSP).

**Table 4:** Effect of tamarind seed powder (TSP) on phytochemical composition of cookies

TSP concentration	TPC (mg GAE 100 mg^−1^)	TFC (mg CE 100 mg^−1^)	TAA (mg VCE 100 mg^−1^)	TTC (mg CE 100 mg^−1^)
Control	20.43 ± 0.29^a^	4.06 ± 0.06^a^	5.6 ± 0.01^a^	8.3 ± 0.73^a^
2.0% TSP	23.41 ± 0.31^b^	5.35 ± 0.07^b^	8.9 ± 0.07^b^	11.70 ± 1.06^b^
4.0% TSP	25.37 ± 0.20^c^	5.71 ± 0.05^c^	12.7 ± 0.08^c^	12.35 ± 0.5^c^
6% TSP	26.1 ± 0.05^d^	6.67 ± 0.29^d^	17.2 ± 0.06^d^	13.54 ± 0.27^d^
8% TSP	27.41 ± 0.09^e^	8.2 ± 0.08^e^	19.2 ± 0.03^e^	15.48 ± 0.43^e^
10% TSP	29.08 ± 0.23^f^	10.29 ± 0.07^f^	25.5 ± 0.04^f^	19.24 ± 0.40^f^

TPC, total phenolic content; TFC, total flavonoid content; TAA, total antioxidant activity; TCT, total condensed tannins. Data are means ± standard deviation from three independent experiments (*n* = 3). Mean values in the same column with different superscript letters are significantly different (*P* < 0.05).

**Figure 2:**
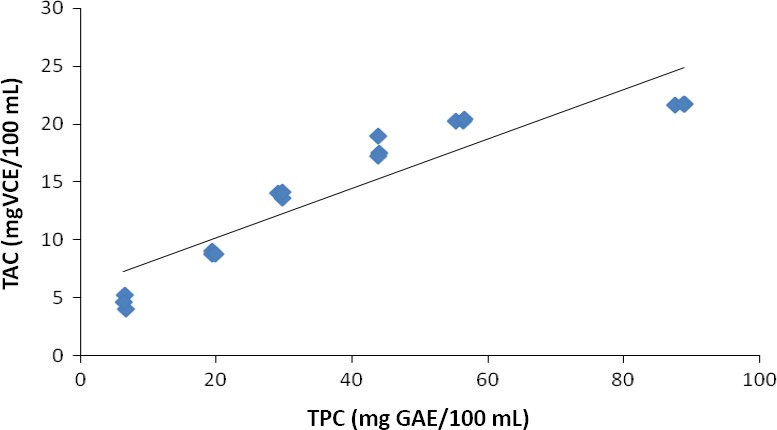
Relationship between antioxidant activity and total phenolic content mango juice enriched with TSP (*R* ^2^ =^ ^0.922).

There was a significant positive correlation between total phenolic content (TPC) and total antioxidant activity (TAC) (*R* ^2 ^=^ ^0.922, *P* < 0.05) as shown in Figure 2.

**Figure 3:**
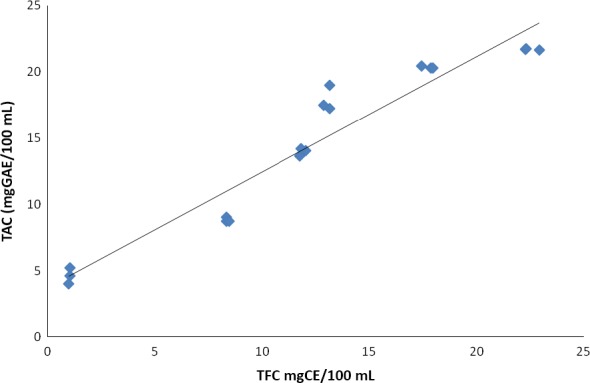
Relationship between antioxidant activity and total flavonoid content of mango juice enriched with TSP (*R* ^2 ^= 0.923).

There was a significant positive correlation (*R* ^2^ = 0.923, *P* < 0.05) between TAC and TFC as shown in Figure 3.

### Effect of tamarind seed powder on sensory acceptability of enriched mango juice

The acceptability scores of all sensory attributes lowered with increase in concentration of tamarind seed powder in enriched mango juice (Table 5). Mango juice enriched with 2.5% TSP received the lowest perceived scores in all sensory attributes (Table 5). The general acceptability of the enriched juice ranged from 4.9 to 8.1. Overall Sensory attributes of 0.5%, 1%, and 1.5% mango–tamarind seed juices were well accepted with a score between 6.6 and 7 (like moderately). Addition of TSP up to 0.5% negatively affected all sensory attributes apart from thickness. Majority the of panelists mentioned that 2.5% TSP‐enriched mango juices was more viscous/thicker than 0%, 0.5%, 1.0%, 1.5% and 2%. Some panelists also noted that 2.5% TSP‐enriched mango juice was more astringent in flavor compared to the other juices. There was no significant difference (*P* < 0.05) between 0.5%, 1.0% and 1.5% TSP‐enriched mango juice in terms of flavor, taste, consistency, sweetness, and overall acceptability.

**Table 5:** Effect of tamarind seed powder (TSP) on sensory acceptability of enriched mango juice

Attribute	Inclusion level of TSP (%)					
	0 (Control)	0.5	1.0	1.5	2.0	2.5
Appearance	7.9 ± 1.88^a^	6.9 ± 1.37^bc^	6.8 ± 1.27^b^	6.5 ± 1.24^c^	6.1 ± 1.48^cd^	5.9 ± 1.93^d^
Color	8.0 ± 1.71^a^	7.0 ± 1.07^b^	6.7 ± 1.27^b^	6.5 ± 1.29^cb^	6.0 ± 1.51^c^	6.1 ± 1.82^c^
Flavor	7.9 ± 1.09^a^	6.5 ± 1.50^b^	6.0 ± 1.93^b^	5.9 ± 1.87^b^	5.2 ± 1.97^c^	4.9 ± 2.14^c^
Taste	7.7 ± 1.41^a^	6.4 ± 1.41^b^	6.2 ± 1.81^b^	5.8 ± 1.93^b^	4.9 ± 2.07^c^	4.8 ± 2.12^c^
Thickness	6.7 ± 2.03^ab^	6.7 ± 1.76^ab^	6.9 ± 1.57^a^	6.0 ± 1.85^b^	6.3 ± 1.82^ab^	5.9 ± 2.42^b^
Consistency	7.2 ± 1.67^a^	6.4 ± 1.33^b^	6.4 ± 1.86^b^	6.4 ± 1.50^b^	5.8 ± 1.85^bc^	5.4 ± 1.89^c^
Mouth feel	7.4 ± 1.59^a^	6.3 ± 1.88^bc^	6.3 ± 1.77^c^	6.0 ± 1.95^b^	5.3 ± 2.24^db^	5.2 ± 2.20^d^
Sweetness	7.7 ± 1.49^a^	6.3 ± 1.69^b^	6.1 ± 1.93^b^	5.9 ± 2.27^bc^	5.2 ± 2.16^c^	4.9 ± 2.26^c^
Overall acceptability	8.1 ± 1.03^a^	7.0 ± 1.46^b^	6.7 ± 1.61^b^	6.6 ± 1.99^b^	5.3 ± 2.02^c^	4.9 ± 1.98^c^

Values are means ± standard deviation (*n* = 50). Mean values in the same row with different superscripts (a–d) are significantly different (*P* < 0.05). Anchors for the hedonic scale used were: 9 = like extremely, 8 = like very much, 7 = like moderately, 6 = like slightly, 5 = neither like nor dislike, 4 = dislike slightly, 3 = dislike moderately, 2 = dislike very much, 1 = dislike extremely.

Correlation of overall acceptability with taste (*r* = 0.744), mouth feel (*r* = 0.738), sweetness (*r* = 0.783) and flavor (*r* = 0.682) showed that each of the properties is of high significance (*P* < 0.01) in determining the acceptability of the TSP‐enriched mango juice (Table 6).

**Table 6:** Correlation coefficient of the acceptability selected sensory attributes and general acceptability

Attribute	Flavor	Taste	Consistency	Mouthful	Sweetness	Overall acceptability
Flavor	1	0.758^a^	0.464^a^	0.552^a^	0.603^a^	0.682^a^
Taste	0.758^a^	1	0.528^a^	0.624^a^	0.707^a^	0.744^a^
Mouth feel	0.552^a^	0.624^a^	0.579^a^	1	0.710^a^	0.738^a^
Sweetness	0.603^a^	0.707^a^	0.485^a^	0.710^a^	1	0.783^a^

a *P* < 0.01.

### Effect of tamarind seed powder on sensory acceptability of enriched cookies

The scores of all sensory attributes lowered with increase in concentration of tamarind seed powder in enriched cookies (Table 7).

**Table 7:** Effect of tamarind seed powder (TSP) on sensory acceptability of enriched cookies

Attribute	Inclusion level of TSP (%)					
	0 (Control)	2.0	4.0	6.0	8.0	10
Appearance	7.7 ± 1.33^a^	7.6 ± 0.90^a^	6.8 ± 1.61^b^	6.6 ± 1.31^bc^	6.8 ± 1.37^b^	6.2 ± 1.50^c^
Color	7.4 ± 1.5^a^	7.4 ± 1.06^a^	6.8 ± 1.53^b^	6.6 ± 1.45^b^	6.8 ± 1.36^b^	6.0 ± 1.51^c^
Flavor	7.6 ± 1.19^a^	7.5 ± 1.20^a^	7.1 ± 1.52^ab^	6.7 ± 1.29^b^	6.4 ± 1.58^c^	6.1 ± 1.14^c^
Taste	7.5 ± 1.40^a^	7.4 ± 1.19^a^	7.0 ± 1.33^ab^	6.6 ± 1.40^bc^	6.6 ± 1.38^bc^	6.3 ± 1.70^c^
Texture	8.4 ± 6.03^a^	7.2 ± 1.20^b^	7.3 ± 1.30^b^	6.7 ± 1.11^b^	7.1 ± 1.30^b^	6.6 ± 1.50^b^
Mouth feel	7.3 ± 1.51^a^	7.2 ± 1.26^ab^	7.1 ± 1.16^ab^	6.8 ± 1.49^ab^	6.9 ± 1.35^ab^	6.6 ± 1.89^b^
After taste	7.3 ± 1.45^a^	7.0 ± 1.59^ab^	6.8 ± 1.42^abc^	6.5 ± 1.50^bc^	6.8 ± 1.34^abc^	6.4 ± 1.48^c^
Crunchiness	7.7 ± 1.37^a^	7.6 ± 1.39^a^	7.7 ± 0.92^a^	7.5 ± 1.06^ab^	7.02 ± 1.41^b^	7.1 ± 1.45^b^
Overall acceptability	7.8.1 ± 1.83^a^	7.7 ± 0.90^ab^	7.3 ± 1.15^bc^	7.0 ± 1.07^cd^	6.9 ± 1.03^cd^	6.6 ± 1.47^d^

Data are means ± standard deviation (*n* = 50). Mean values in the same row with different superscript letters are significantly different (*P* < 0.05). Anchors for the hedonic scale used were: 9 = like extremely, 8 = like very much, 7 = like moderately, 6 = like slightly, 5 = neither like nor dislike, 4 = dislike slightly, 3 = dislike moderately, 2 = dislike very much, 1 = dislike extremely.

The results show that the addition TSP in cookie formulation resulted in lower scores in all sensory attributes at high concentrations with an overall acceptability ranging from 6.6 ± 1.47 to 7.8.1 ± 1.83. Addition of TSP up to 2.0% did not affect acceptability for appearance, color, flavor, crunchiness and taste of the enriched cookies (Table 7). Addition of tamarind seed powder above 8% significantly (*P* < 0.05) reduces the acceptability for color of the enriched cookies. Incorporation of tamarind seed powder significantly affected texture in relation to the control. In terms of overall acceptability tamarind seed powder‐enriched cookies were acceptable up to 10% tamarind seed powder (6.6 = like slightly). Although, 6% tamarind seed powder‐enriched cookie was not significantly different from 8% tamarind seed‐enriched cookie in terms of overall acceptability, it differed in terms of flavor.

## Discussion

### Effect of tamarind seed on physicochemical properties of mango juice and cookies

Addition of tamarind seed powder led to a significant increase (*P* < 0.05) in pH of mango juice. Tamarind seed powder has a pH 5.2 ± 0.01 (Oluseyi and Temitayo 2015) and therefore may be responsible for the increase of the pH of the enriched mango juice that was observed. The reduction of total titratable acidity is consistent with the increase in pH of the enriched mango juices. The reduction in titratable acidity may be attributed to tamarind seed powder since there was increase in pH with increase in tamarind seed powder concentration. The low titratable acidity and high pH are associated with reduction in shelf life. Acidulants like citric acid should be added to tamarind seed powder‐enriched mango juice to increase its shelf life (Pundhir and Murtaza 2015).

Results of this study revealed that addition of tamarind seed powder led to a concentration‐dependent increase in mango juice viscosity. These results are consistent with observations by Kumar and Bhattacharya (2008) who studied the rheological behavior of Tamarind Kernel Powder (TKP) of varying concentration (2, 4, 6, 8, and 10%) and observed an increase in apparent viscosity with increase in TKP concentration. The increment in viscosity of the enriched mango juices may be attributed to large proportions (65.1–72.2%) of galactoxyloglucan found in tamarind seed (Bhattacharya et al. 1994). Galactoxyloglucan is composed of a *β*‐ (1–4) linked D‐ glucan backbone that is substituted with side‐chains of *α*‐d‐xylopyranose and *β*‐d‐galactopyranosyl (1‐2)‐*α*‐d‐xylopyranose linked to (1–6) glucose residue (Yamanaka et al. 2000). Galactoxyloglucan exhibits high water‐holding capacity with good stability to heat, acids, and shear and thus its wide application in the food industry as a thickener, stabilizer, or starch modifier. It is used to improve rheological and thermal properties of many products including, salad dressing, mayonnaise, and stew (Nishinari et al. 2000). This therefore implies that tamarind seed powder can potentially be utilized as a food stabilizer.

The coloration of the enriched juices became more orange and dull with increased concentration of tamarind seed powder. A similar observation was made by Andabati and Muyonga (2014) where addition of tamarind seed powder to the tamarind pulp juice led to discoloration of the enriched juices. This may be attributed to the activity of polyphenol oxidase. TSP is rich in polyphenols which are substrates for the polyphenol oxidases. In presence of oxygen, the enzymes catalyze the hydroxylation of monophenols to diphenols and then subsequently to corresponding quinine intermediates. It is the intermediates that are responsible for discoloration. More so, TSP has a dark brownish color (Kumar and Bhattacharya 2008), and therefore incorporation of TSP in mango juice decreases its brightness. Color is very important when consumers are making choices on food products (Tril et al. 2014). It is therefore important to use low concentration of TSP in juices to avoid discoloration/darkening of juices which potentially has an effect on consumer preference. High concentrations of TSP can be tried in other products such as sausages whose color may not be affected (Tril et al. 2014).

### Effect of TSP on phytochemical composition of enriched mango juices and cookies

The effect of TSP on phytochemical content of enriched mango juice and cookies was concentration dependent. In their studies Andabati and Muyonga (2014) found that addition of tamarind seed powder in tamarind pulp juice led to a significant enrichment in total phenolic compounds, total flavonoids and total antioxidant activity. The observed increase in TFC, TPC and TAA in enriched juices is attributed to the high phenolic content, flavonoid content, and antioxidant activity in *Tamarindus indica* L. seeds (Tsuda et al. 1994; Soong and Barlow 2004; Siddhuraju 2007). A similar observation was reported by Salgado et al. (2012) where addition of antioxidant‐rich pomegranate peel (*Punica granatum*) extract to orange and tomato juice led to increase in antioxidant activity. The total phenolic content of TSP‐enriched mango juices was in the range 6.54 ± 0.21 to 88.44 ± 0.8 mg GAE/100 mL. This is higher than the phenolic content in tomato juice (5.97 mg GAE/100 mL) (Owusu et al. 2015), passion fruit (27.1–38.1 mg GAE/100 g) (Ramaiya et al. 2013), mango (6.25 mg GAE/100 g) (Gorinstein et al. 1999), jackfruit 0.36 mg GAE/100 g (Swami et al. 2012) and fruit juices enriched with roselle fruit (53.7‐10.8 GAE mg/100 g) (Mgaya et al. 2014). There was a 13‐fold increase in the total phenolic content at 2.5% of tamarind seed powder‐enriched juice compared to the control. This suggests that addition of tamarind seed powder highly enhances the content of bioactive compounds, thus increasing the nutraceutical properties of the enriched mango juices. It is therefore important to further utilize the seeds rather than just discarding them as waste as it is a common practice currently. Flavonoid intake of about 14.33 mg/day has been reported to reduce memory loss in elderly people (Letenneur et al. 2007). Daily consumption of 110 mL of 1.5% TSP‐enriched mango juice is adequate to meet flavonoid content of 14.33 mg/day. Therefore, the findings from the current study show the potential of tamarind seed‐enriched mango juices in mitigating memory loss in elderly people.

Antioxidant activity is very important in human health mainly because of its free‐radical scavenging activity and protection against oxidative stress (Haghju and Almasi 2015; Valdes et al. 2015) and thus prevents development of diseases such as heart disease and cancer (González‐Vallinas et al. 2013; Farias et al. 2014)**.** Numerous studies have demonstrated that polyphenol and flavonoid compounds are the most effective antioxidative constituents in fruits, vegetables and grains (Choi et al. 2007; Dykes and Rooney 2007). This is consistent with significant positive correlation between total antioxidant activity and the concentrations of total phenolics and total flavonoid in tamarind seed‐enriched juices observed in this study. Similar relations have been reported for other foods such as Chilean blackberries, barley, mushrooms, mulberries, flaxseed, wheat, oats, ginseng rice, and bread enriched with ginger (Choi et al. 2007; Céspedes et al. 2008; Jayaprakasha et al. 2008; Shen et al. 2009; Balestra et al. 2011).

The addition of tamarind seed powder to mango juices increased the content of total phenolics, flavonoid, antioxidant activity, and tannin in the enriched cookies in a concentration‐dependent manner. In a related study Ajila et al. (2008) observed that incorporation of mango peel powder (0, 5, 10, 15, 20%) increased total phenolics and antioxidant activity in a concentration‐dependent manner. The assay of DPPH‐scavenging activity showed that TSP was a good source of active compounds, and adding it significantly enhanced the antioxidant properties of the cookies. A similar study Mildner‐Szkudlarz et al. (2013) reported that incorporation of white grape pomace (residue) significantly increased the content of phenolics and antioxidants in biscuits.

The beta carotene levels decreased with increase in tamarind seed powder concentration in enriched juices. Addition of TSP may have contributed to dilution of beta carotene content. However, it was added in quite small quantities up to 2.5% to account for all the change. Another reason is probably formation of the complexes between the proteins in the seed powder with carotenoids in the enriched mango juices. This reduces carotenoid concentration in juice with increase in tamarind seed (Sweeney and Marsh 1971).

### Effect of tamarind seed powder on sensory properties of enriched products

Addition of tamarind seed powder in mango juice resulted in reduced scores in all the sensory attributes including color, flavor, consistency, thickness, and general acceptability. In a related study, Salgado et al. (2012) observed low scores when pomegranate peel powder was added to orange and tomato juice. Both powders have high phenolics and tannins which elicit undesired astringent taste (Kumar and Bhattacharya 2008; McRae and Kennedy 2011). Phenolic compounds highly correlate inherently with sensory characteristics of food, such as color, astringency, bitterness, and flavor (Mousavinejad et al. 2009). High levels of phenolics and tannins can elicit negative consumer reactions (Drewnowski and Gomez‐Carneros 2000). Astringency is the drying, roughing, and puckering of the epithelium of the oral cavity. The perception of astringency results from binding and subsequent precipitation of tannins with salivary proteins and glycoproteins. This interaction acts as a water barrier resulting in a tactile sensational loss of lubrication in the oral cavity (Kielhorn and Thorngate III 1999). This therefore explains the reduction in scores in sensory acceptability with increase in concentration of TSP in the current study. The lowest score on the sensory attributes was recorded at the highest concentration of 2.5% of the TSP in mango juice. According to Lawless et al. (2012), addition of high level of polyphenol compounds in food formulations negatively affects the sensory attributes and acceptability of finished foods, resulting in changes such as increased bitterness and astringency. The sensory acceptability results of this study confirm the association of astringency to poor acceptability. The low consumer acceptance ratings for the thickness and flavor of the enriched juices scores were substantiated by the findings on viscosity and phytochemical analysis findings in the current study. There was no significant difference in acceptance for flavor, taste, thickness, and overall acceptability between 0.5%, 1.0%, 1.5%, respectively, while at 2.0% and 2.5% there was a significant difference of the enriched juices. The results of this study show that juice with 2% and 2.5% TSP had significantly lower acceptability compared to the control and those with lower TSP concentrations. This therefore suggests that 1.5% is the maximum concentration possible that can be added to enrich mango juice that can have commercial appeal because it was the acceptance limit of sensory evaluation by the tasters.

While consumers may be interested in products with high levels of bioactive products, palatability and taste are key determinants of acceptability. This, therefore, suggests that a concentration of 1.5% of TSP would be an appropriate level to cater for consumer acceptability as well as providing health benefits of phenolics.

Incorporation of tamarind seed powder into cookies resulted in reduced scores in all the sensory attributes. Sensory evaluation studies showed flavor, color, taste, crunchiness, and overall general acceptability of cookies containing tamarind seed were as acceptable as those of control cookies up to 2% level of tamarind seed incorporation and any further increase led to lower scores. The enriched cookies also became relatively harder compared to the control. Tamarind seed powder contains galactoxylose which has high water‐binding capacity (Bhattacharya et al. 1994) and this may explain the hardness of the enriched cookies. The addition of TSP influenced the color of each of the cookies. In the cookies made with 10% TSP panelists commented on the unappealing dark color. This may be due to the brown of the tamarind seed (Kumar and Bhattacharya 2008). These comments were reflected in the significantly lower acceptance scores for enriched cookies color compared with the control.

At higher TSP concentration, the acceptability for mouth feel and after taste scores was also low. It is known that the polyphenolic compounds contribute to the astringency of enriched cookies because of the interaction between phenolics, mainly procyanidins and the glycoproteins in saliva (McRae and Kennedy 2011). The tamarind seed has high phenolics and tannins which elicit undesired astringent taste (Kumar and Bhattacharya 2008) which makes products with tamarind seed powder have lower consumer appeal. Based on all these sensory attributes, participants preferred a control cookie, rather than enriched cookies. This is consistent with the study by Bakke and Vickers (2011), in which, the bitterness from added wheat germ extract decreased bread liking. In terms of overall acceptability, cookies enriched with (6%, 8%, 10%) TSP were not significantly different. However, 8% and 10% TSP‐enriched cookies had significantly lower scores for flavor (6.4) and color (6.0), respectively. On that basis, the 6% TSP‐enriched cookie was selected as the maximum acceptable TSP concentration for TSP‐enriched cookies.

Other efforts have been made to add bioactive‐rich components to processed foods. (Ajila et al. (2008); Hooda and Jood (2005); Mildner‐Szkudlarz et al. (2013)) added mango peel powder, fenugreek, and white grape pomace powder to biscuits and reported no negative effect with levels of up to 10% (Sudha et al. 2007) added apple pomace to biscuits and found that up to 20% could be incorporated without compromising acceptability.

## Conclusion

Incorporation of the tamarind seed powder into mango juice and cookies significantly increases their content of bioactive phytochemicals with an associated increase in the antioxidant activity. Based on sensory analysis, it may be concluded that the amount of TSP that can be added to mango juice and cookies need to be limited to 1.5% and 6%, respectively, to ensure consumer acceptability. The findings confirm the potential to utilize tamarind seed powder as a source of natural antioxidants and stabilizer in our search for good human health.

## Conflict of Interest

The authors did not declare any conflict of interest.
